# Application of Single Molecule Fluorescence Microscopy to Characterize the Penetration of a Large Amphiphilic Molecule in the Stratum Corneum of Human Skin

**DOI:** 10.3390/ijms16046960

**Published:** 2015-03-27

**Authors:** Pierre Volz, Alexander Boreham, Alexander Wolf, Tai-Yang Kim, Jens Balke, Janna Frombach, Sabrina Hadam, Zahra Afraz, Fiorenza Rancan, Ulrike Blume-Peytavi, Annika Vogt, Ulrike Alexiev

**Affiliations:** 1 Department of Physics, Institute of Experimental Physics, Freie Universität Berlin, Arnimallee 14, 14195 Berlin, Germany; E-Mails: pierre.volz@fu-berlin.de (P.V.); alexander.boreham@fu-berlin.de (A.B.); alexander.wolf@fu-berlin.de (A.W.); tai-yang.kim@fu-berlin.de (T.-Y.K.); jens.balke@fu-berlin.de (J.B.); 2 Clinical Research Center for Hair and Skin Science, Department of Dermatology, Charité-Universitaetsmedizin Berlin, Charitéplatz 1, 10117 Berlin, Germany; E-Mails: janna.frombach@charite.de (J.F.); sabrina.hadam@charite.de (S.H.); zahra.afraz@charite.de (Z.A.); fiorenza.rancan@charite.de (F.R.); ulrike.blume-peytavi@charite.de (U.B.-P.)

**Keywords:** single molecule fluorescence microscopy, total internal reflection fluorescence microscopy, single particle tracking, skin penetration, penetration pathways, stratum corneum, tape stripping

## Abstract

We report here on the application of laser-based single molecule total internal reflection fluorescence microscopy (TIRFM) to study the penetration of molecules through the skin. Penetration of topically applied drug molecules is often observed to be limited by the size of the respective drug. However, the molecular mechanisms which govern the penetration of molecules through the outermost layer of the skin are still largely unknown. As a model compound we have chosen a larger amphiphilic molecule (fluorescent dye ATTO-Oxa12) with a molecular weight >700 Da that was applied to excised human skin. ATTO-Oxa12 penetrated through the stratum corneum (SC) into the viable epidermis as revealed by TIRFM of cryosections. Single particle tracking of ATTO-Oxa12 within SC sheets obtained by tape stripping allowed us to gain information on the localization as well as the lateral diffusion dynamics of these molecules. ATTO-Oxa12 appeared to be highly confined in the SC lipid region between (intercellular space) or close to the envelope of the corneocytes. Three main distinct confinement sizes of 52 ± 6, 118 ± 4, and 205 ± 5 nm were determined. We conclude that for this amphiphilic model compound several pathways through the skin exist.

## 1. Introduction

Treatment of skin disorders by topical therapy, *i.e.*, topical application of active molecules on the skin surface, is challenging because the stratum corneum (SC) represents an effective physical and biochemical barrier [1]. In fact, molecular weight is a major determinant of penetration. Furthermore, the possibility to enter the skin depends on the polarity and lipophilicity of the applied compound. The observation that molecules >500 Da usually do not reach therapeutic concentrations is frequently referred to as the “500 Dalton rule” [2]. Yet, some therapeutic effects can be observed even with larger molecules such as the macrolide lactone tacrolimus (822 Dalton), which is an immunosuppressive drug used, for instance, in the treatment of atopic dermatitis [3]. Also, increasing experimental evidence suggests that large molecules can be recognized by the skin immune system through scanning and uptake activities of dendrites, which occur right below the SC [4]. A better understanding of how larger compounds cross the SC could help to develop strategies to facilitate penetration.

**Figure 1:**
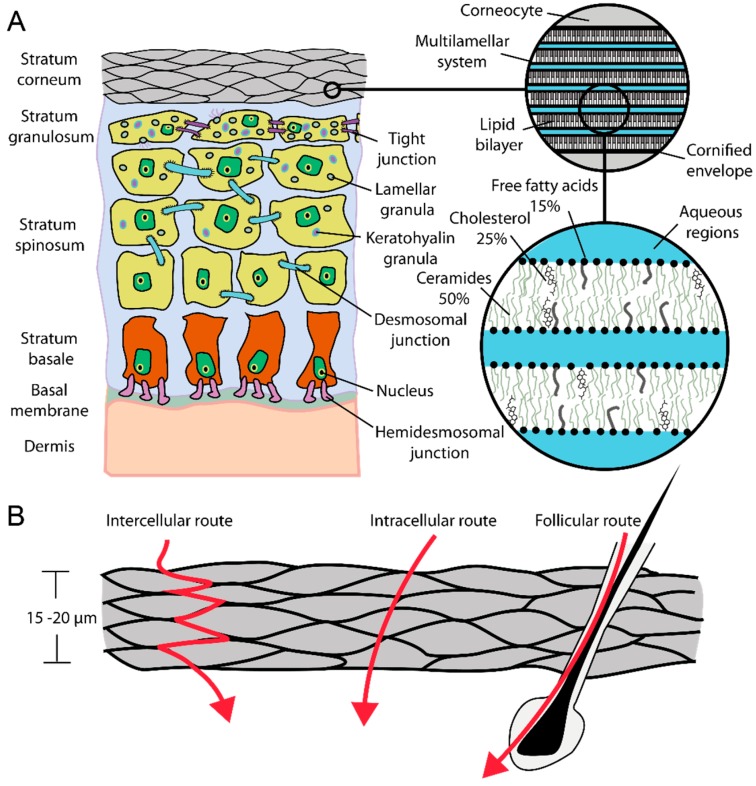
(**A**) Schematic of skin; (**B**) Dermal penetration routes.

The outermost layer of the skin, the SC (Figure 1A,B), is the main physical barrier of the skin. In the SC typically 15–20 cell layers of nonmetabolical corneocytes are embedded in a lipid matrix consisting mainly (about 90% of the total lipid content) of ceramides, cholesterol, and free fatty acids [5]. Due to the strong flattening of the corneocytes the human SC is 15–20 μm thick. A simple model to describe the stratum corneum is the brick-mortar-model by Elias [6]. Here, the bricks are associated with corneocytes, which provide for mechanical and chemical stability via their keratin filament network, while the extracellular lipid matrix represents the mortar, which helps to seal the skin against harmful agents such as pathogens. The thickness of the viable epidermis underneath the SC (Figure 1A) varies in dependence on the body site and is typically between 50 and 100 μm [7,8,9]. Keratinocytes are the most common cell type within the viable epidermis. This cell type undergoes a transformation process while migrating from the lowest layer of the epidermis, called stratum basale, towards the SC. Single keratinocytes are connected by desmosomal junctions. Additional hemidesmosoms mediate the cell contact to the basal membrane, a thin layer separating dermis and epidermis. The basal membrane is crucial for the regulation of the cell and nutrition transport into the non-vascular epidermis [10,11].

For the penetration of molecules through the SC three main pathways have been suggested: the intercellular, intracellular (transcellular), and the transfollicular route (Figure 1B). While Franz diffusion cell experiments and whole tissue extracts only help to determine overall penetration rates and drug concentrations in the different tissue layers [12], high resolution imaging techniques are required to address the question of whether deep penetration occurs and which pathways the molecules take. Several different techniques have been employed in the past to characterize SC penetration. Techniques such as neutron scattering, X-ray diffraction, electron microscopy, and advanced fluorescence microscopy methods have been used to characterize the structural and dynamical parameters in the skin [13,14,15,16,17,18,19,20]. Tape stripping together with appropriate analytical methods is frequently applied to quantify penetration depths along the SC [21,22,23,24]. An adhesive tape strip is pressed onto the treated skin area and then removed. This procedure is repeated several times and enables the analysis of the SC layer by layer. Analytical methods range from invasive (destruction of removed SC layers) to non-invasive methods, such as optical techniques. Laser scanning confocal microscopy, in particular multiphoton fluorescence excitation based techniques, provide the possibility to obtain spatially resolved information of fluorescent probes within the skin at different depth *in vitro* and *in vivo* [24,25,26]. However, dynamic information in terms of local diffusion coefficients and local restrictions in diffusion of penetrating molecules within the SC is not trivial to obtain. The former was recently investigated using a combination of multiphoton fluorescence excitation and raster image correlation spectroscopy (RICS) [26]. Local restrictions in diffusion of single molecules with a positional accuracy down to some tens of nanometers can be detected using single molecule microscopy [27], information not available from confocal laser scanning microscopy.

This is important when focusing on the different penetration pathways that are determined by the structure and dynamics of the lipid matrix, e.g., the intercellular route. Structural considerations of the lipid multilayer assembly in the intercorneocyte matrix (Figure 1A) suggest that lipophilic pathways exist, based on the repeat distances in the lamellar lipid phases of approximately 6 and 13 nm, in which molecules smaller than 5–10 nm have chances to diffuse [10,15].

Aqueous pores also have been suggested to exist in between the lipid head groups of the stacked lipid bilayers and are referred to as the polar route. It is believed that predominantly polar molecules penetrate via this route and it is speculated that the dimensions may vary from ≤30 to 100 nm [28], depending on the localization in the intercorneocyte space or in the broader intercluster region between clusters of 3–10 corneocytes [1].

While single molecule microscopy can be applied to living transparent organisms such as zebrafish embryos [27], studying fluorescent molecules in turbid tissue, such as skin, at the single molecule level is challenging. However, it has to be noted, that single fluorescently labeled nanoparticles were visualized in the skin using multiphoton microscopy [29].

Since the tape stripping procedure allows intact thin layers of the stratum corneum to be obtained [23,30], we combined this technique with total internal reflection fluorescence microscopy (TIRFM). TIRFM is sensitive only to fluorescent molecules within ~200 nm distance to the coverslip surface and thereby restricts the background fluorescence and increases the signal-to-noise ratio in the resulting images and hence single molecules can be detected. The sensitivity within ~200 nm distance nicely matches the average thickness of a single corneocyte with about 200–300 nm [10,16]. To the best of our knowledge single molecule TIRFM has not been applied before to skin. Thus, we describe here the application of single molecule TIRFM and single particle tracking (SPT) to localize and follow the lateral diffusion of molecules within different depths of the SC that were accessed by serial tape stripping. The localization of fluorescent molecules within the SC layers can be resolved down to 10 nm by single molecule TIRFM. As a penetrating model compound we used the amphiphilic molecule ATTO-Oxa12, a commercially available fluorescent dye [31], with a molecular weight of 835 Da, larger than the 500 Da cut-off.

## 2. Results and Discussion

### 2.1. Detection of the Amphiphilic ATTO-Oxa12 Molecule in Cryosections of Excised Skin

**Figure 2:**
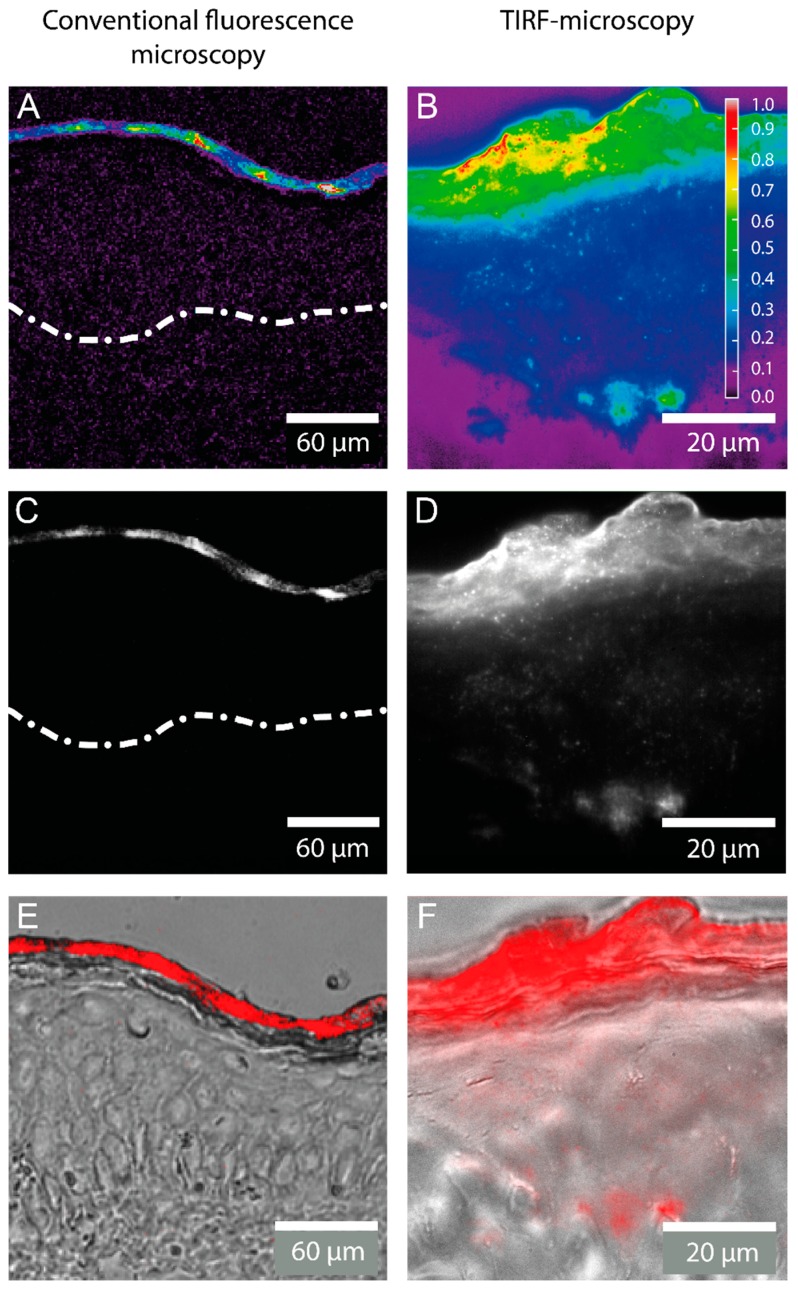
Fluorescence microscopy images of a cryosection through excised human skin with ATTO-Oxa12 applied topically as obtained by conventional fluorescence microscopy and total internal reflection fluorescence (TIRF) microscopy. False color coded images representing the relative fluorescence intensity are shown in (**A**,**B**); while (**C**,**D**) display grayscale intensity images. The white spots in (**D**) indicate ATTO-Oxa12 molecules in the stratum corneum (SC) and the viable epidermis; (**E**,**F**) are digital overlays of fluorescence microscopy images (here red) from (**C**,**D**) with bright-field microscopy images of the skin. The broken line in (**A**,**C**) indicates the barrier between the viable epidermis and the dermis.

Conventional fluorescence microscopy of cryosections obtained from human skin samples treated with the positively charged, lipophilic fluorescent dye ATTO-Oxa12 suggested that a vast amount of substance remained on the skin surface with no or minimal penetration to deeper layers (Figure 2C,E). The digital overlay in Figure 2C shows strong fluorescence in the SC, but deeper layers such as the viable epidermis seem to be unstained. Figure 2B,D,F displays the corresponding TIRFM images. As illustrated there, TIRF microscopy of longitudinal sections of human skin confirmed the presence of ATTO-Oxa12 molecules throughout the SC, in the subcorneal compartments and viable epidermis. In accordance with the results from conventional microscopy the highest concentration of the dye ATTO-Oxa12 was found in the SC (Figure 2E,F).

### 2.2. Stratum Corneum (SC) Profiles from Excised Skin

**Figure 3:**
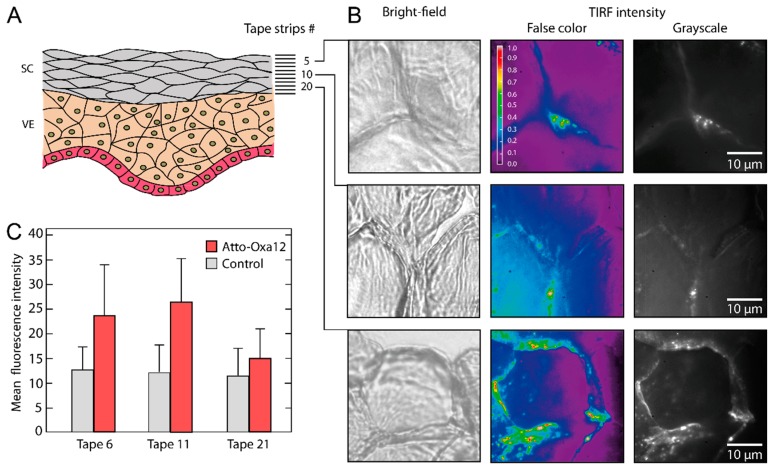
(**A**) Schematic of stratum corneum (SC) and viable epidermis (VE) with indicated tape strip numbers; (**B**) False color coded images representing the relative fluorescence intensity and grayscale TIRFM images of tape 5, 10, and 20 are shown together with the corresponding bright-field microscopy images; (**C**) Mean fluorescence intensity as obtained from tapes 6, 11, and 21 with conventional fluorescence microscopy.

SC profiles obtained by serial tape strippings are frequently applied to quantify penetration depths along the stratum corneum [22,23]. Yet, as substantial loss of material occurs during the elution process, a correlation with SC compartments is not possible. Direct fluorescence microscopy of tapes gives some orientation with regard to the presence of dye. The mean fluorescence intensity values obtained from these images (Figure 3C) are characterized by large errors and only give a superficial impression of the penetration depth. However, it has to be noted that in these experiments the fluorescence excitation wavelength was not optimal for the used dye (see Experimental Section). Nevertheless, lack of spatial resolution, low dye concentrations in deeper layers of the stratum corneum, as well as tape autofluorescence, limit the value of conventional microscopy.

**Figure 4:**
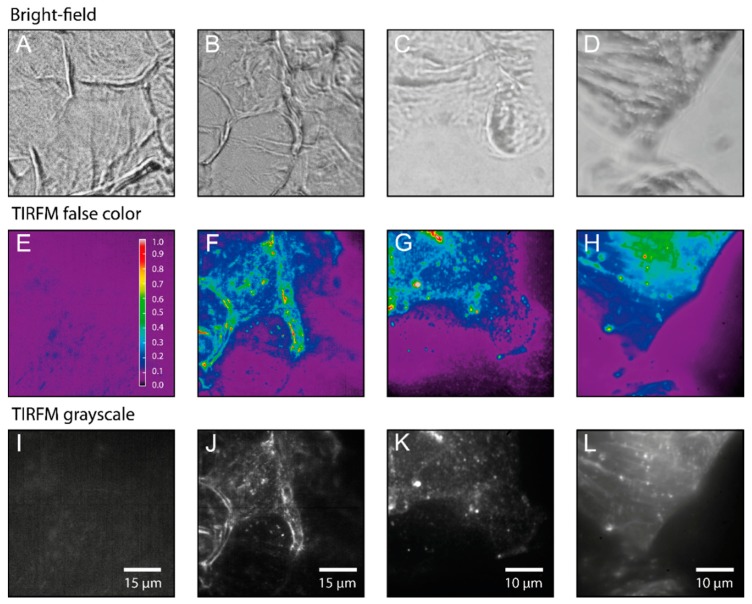
Bright-field microscopy (**A**–**D**), false color coded images representing the relative fluorescence intensity (**E**–**H**) and grayscale TIRFM images (**I**–**L**). (**A**,**E**,**I**) Control sample (unstained) from tape 5. (**B**–**D**,**F**–**H**,**J**–**L**) Three different ATTO-OXA12 samples from tape 5/6. TIRF Images were taken with a CMOS (complementary metal-oxide semiconductor) camera (**E**,**F**,**I**,**J**) and EMCCD (electron multiplying charge coupled device) camera (**G**,**H**,**K**,**L**). Measurement conditions are as described in the Experimental Section.

Tape strips of thin SC layers—a single flat corneocyte is about 0.2–0.8 µm thick [10,16]—allowed us to detect the dye ATTO-Oxa12 at the single molecule level with high sensitivity and high specificity using the TIRF mode of a wide-field microscope. TIRF intensity images (Figure 3B) obtained at different depths of the SC (tape 5, 10 and 20) revealed a predominant localization of the dye molecules in the intercellular spaces. We also observed ATTO-Oxa12 molecules close to the envelope of the corneocytes, less frequently in the skin preparation shown in Figure 3B but more frequently in another preparation shown in Figure 4F. This heterogeneity in the localizations probably depends on the stripping procedure and on the properties of the excised skin. In accordance with observations on cryosections (Figure 2) ATTO-Oxa12 is clearly visible in tape 20 using TIRFM (Figure 3B).

### 2.3. Single Particle Tracking of ATTO-Oxa12 Molecules in the SC

Using single particle tracking we were able to analyze the lateral diffusion dynamics of single ATTO-Oxa12 molecules in the different SC layers and to determine the confinement of diffusion. These investigations also gave valuable information on the penetration compartments.

Figure 4E,I illustrates that under the fluorescence excitation conditions used in the TIRFM studies autofluorescence is negligible compared to the ATTO-Oxa12 fluorescence that is clearly visible in the TIRFM images (Figure 4F–H,J–L). The upper row in Figure 4 represents the corresponding bright-field images of the stripped SC layers. In the control sample Figure 4A, as well as in Figure 4B, interconnected corneocytes are visible. In the upper left corner of Figure 4B the typical pentagon-like shape of corneocytes is seen, while another typical shape, a hexagon-like structure, is found in the images presented in Figure 3B. The parallel fluorescent lines in the left part of Figure 4F,J indicate the lateral overlap between two corneocytes (see also Figure 1B) [28]. In Figure 4C,D no clear shape of a single corneocyte is visible. In addition, the quality of the bright-field images differs between Figure 4A,B and Figure 4C,D as the latter were imaged with a lower resolution external camera, while the images in Figure 4A,B were directly recorded with the CMOS camera in the emission path (see Methods Section).

**Figure 5:**
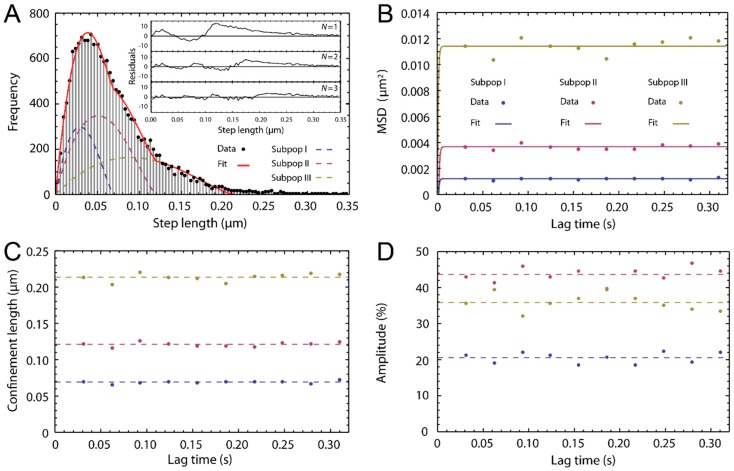
Single particle tracking (SPT) analysis. (**A**) Histogram of the step length distribution for the molecules found in the image presented in Figure 4G (frame lag = 1, *t*_lag_ = 31 ms). The fit with Equation (1), yielding three subpopulations, is shown. The inset shows the residuals for fits with *N* = 1,2,3 subpopulations; (**B**) The mean square displacements (MSD)-lag-time plot of the three subpopulations with data points at ten different lag times and fit with Equation (4); (**C**) Corresponding confinement lengths. The average values are indicated by broken lines; (**D**) Amplitudes of the three subpopulations given in %. The average values are indicated by broken lines.

**Figure 6:**
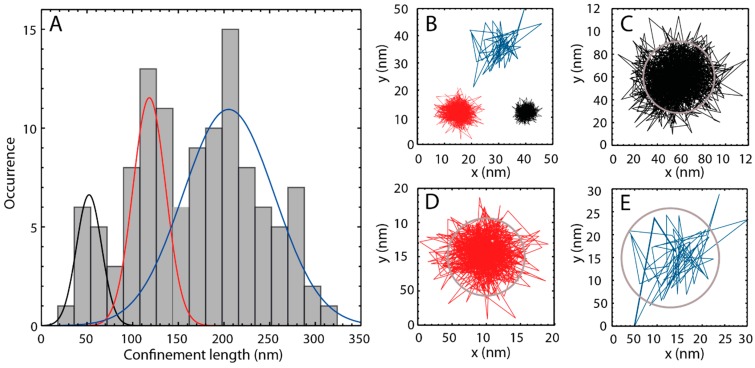
(**A**) Histogram of confinement lengths. The fit represents a multi-Gaussian probability distribution with three subpopulations (subpopulation 1—black line, subpopulation 2—red line, subpopulation 3—blue line); (**B**) Three ATTO-Oxa12 traces, each representative for one of the three subpopulations shown in (**A**). The color of the trace matches the color of the respective subpopulation; (**C**–**E**) Individual ATTO-Oxa12 traces with confinement lengths of 63 ± 3 nm (**C**), 113 ± 5 nm (**D**), and 220 ± 20 nm (**E**). The circular confinements are indicated by gray circles.

To follow and analyze the traces of single ATTO-Oxa12 molecules from the time lapse movies that correspond to the images shown in Figure 4J–L we selected regions of interest where fluorescent spots corresponding to single molecules were detected. For SPT analysis, the coordinates of the fluorescence spots in the frames of the time lapse movies were determined. Single molecule traces were then reconstructed according to the procedure described in the Experimental Section. Representative SPT analysis results are summarized in Figure 5. Single molecule traces (Figure 6B) were analyzed to obtain the mean square displacements (MSD) (Equation (2)). A statistical analysis [32] of the step lengths within the ATTO-Oxa12 traces allowed for the detection of diffusion heterogeneity (Figure 5). The histogram of the step length distribution is best described by a fit with three subpopulations (Equation (1)) as judged by the residuals of fits with different numbers of subpopulations (Figure 5A). These three subpopulations showed confined diffusion behavior, *i.e.*, no linear dependence of the MSD on time, with different limiting values (Figure 5B,C). The fit of the data points presented in Figure 5B with Equation (4) yields confinement lengths of 70.0 ± 0.6, 121 ± 1, and 214 ± 2 nm (Figure 5C). The contribution of the respective subpopulations to the total amplitude of the step length distribution are 21% ± 2%, 43% ± 3%, and 36% ± 3% (Figure 5D).

For comparison with literature values on the diffusion properties of amphiphilic dyes in the SC as measured with multiphoton excitation based RICS at depths between 2–4 µm [26], we quantified the diffusion constant and the confinement lengths of 116 ATTO-Oxa12 molecules from their respective traces in eight different images obtained from tape 5 and 6. Since with tape 10 roughly 30% of the SC is removed and a thickness of the SC of 15–20 µm is assumed, tape 5 would correspond to a SC depth of 2–4 µm.

From Figure 5B it is evident that with a time resolution of 31 ms the linear part of the MSD-time plot was not resolved and data points for the determination of the diffusion coefficient of ATTO-Oxa12 within the confined area are not available. The same holds true for all other experiments with a time resolution of 10 ms. Thus, the limited time resolution only allowed us to determine a lower limit for the diffusion constant from the slope of a linear function that connects the origin with the first available MSD data point (Equation (3)). The diffusion constant of ATTO-Oxa12 in the thin SC layers obtained in this way is *D* = 2.5 ± 1.2 µm^2^/s.

Model studies on SC lipids suggest that polar molecules preferably penetrate along the polar regions of the lipid bilayers that are with 0.5 nm much smaller than the lipophilic channels with about 4–10 nm [6,7,8,15]. Analysis of diffusion constants of fluorescent model compounds within the intact SC using RICS, however, showed no correlation between the diffusion constant and the polarity of the molecule [10], suggesting a more complex penetration mechanism. The diffusion constant of the amphiphilic molecules observed in [21] are 0.83 ± 0.05 µm^2^/s for rhodamine B attached to di-decanoyl-phosphatidylethanolamine and 0.34 ± 0.05 µm^2^/s for the fluorescent dye ATTO647N attached to di-myristoyl-phosphatidylethanolamine. The former value is in the range of the diffusion constant of 2.5 ± 1.2 µm^2^/s obtained here within thin SC layers for ATTO-Oxa12 with single particle tracking. Although the techniques (RICS, single molecule TIRFM) are different and the samples differ, *i.e.*, intact excised skin in the RICS experiments [26] and thin SC layers from excised skin in the single molecule TIRFM experiments, the observation of diffusion constants within the same range support the application of single molecule TIRFM to thin SC layers as a further method to characterize penetration.

In addition to the determination of lateral diffusion coefficients, confinement of diffusion in the nanometer range can be measured by single molecule TIRFM (Figure 5C). Figure 6A shows the histogram of the distribution of the confinement lengths obtained from the 116 ATTO-Oxa12 traces. Clearly, three peaks are visible and the histogram was fitted with a multi-Gaussian probability distribution for three subpopulations. The corresponding confinement lengths are 52 ± 6, 118 ± 4, and 205 ± 5 nm, comparable with the values shown in Figure 5C. The associated peak widths (2σ) are 28 ± 7, 36 ± 6, and 100 ± 12 nm. Occasionally, very narrow confinements were observed with sizes of about 25–46 nm. On the other hand, the largest confinements observed are up to 310 nm. Representative single molecule traces are shown in Figure 6B–E.

The information about confinement lengths is quite important as it gives structural insight into spatial restrictions of diffusion pathways and thus information about penetration pathways. As described above, the confinement values determined for the diffusion of ATTO-Oxa12 in the SC layers are very heterogeneous. We found these heterogeneous confinement values in the intercorneocyte and intercluster space as well as close to the surface of the corneocytes. All values are larger than the proposed width of the lipophilic channels of up to 10 nm. Since the spatial resolution of our setup is about 10 nm, the values obtained for the confinements of ATTO-Oxa12 motion appear to be real confinement sizes. Thus, the smallest main confinement length of 50 nm may be explained by the diffusion of ATTO-Oxa12 within the whole intercorneocyte space of ~75 nm between two corneocytes [33], traversing the lipophilic and polar regions of the multilamellar stacks because of its amphiphatic nature. Following this line of reasoning ATTO-Oxa12 molecules may also reside close to the envelope of the corneocytes to which lipids are covalently bound. Also, the arrangement of corneocytes in larger clusters may lead to pathways with different sizes between these clusters [34], explaining the larger confinements and their heterogeneities. However, since ATTO-Oxa12 is an amphiphilic molecule that is soluble in aqueous solution, it is also possible that the confinement sizes represent the width of polar channels with a dynamically adapted size as suggested in [28].

## 3. Experimental Section

### 3.1. Materials

#### 3.1.1. ATTO-Oxa12

**Figure 7:**
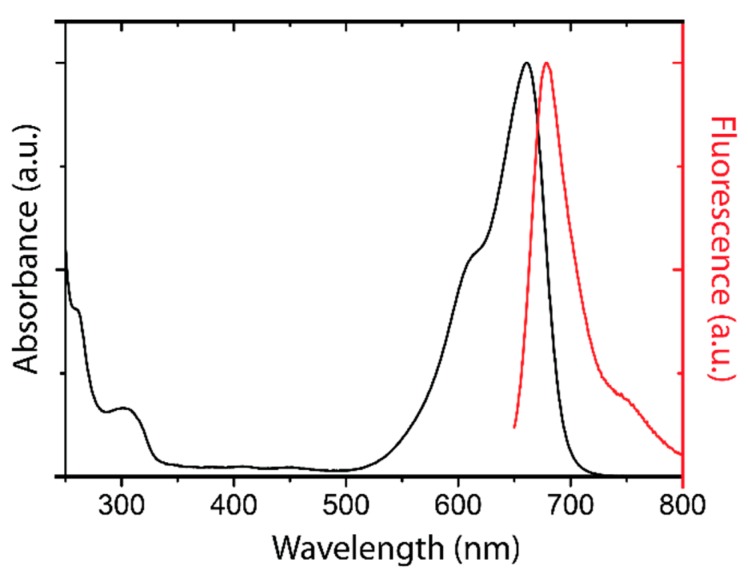
Absorption (black) and fluorescence emission (red) spectrum of ATTO-Oxa12.

The dye ATTO-Oxa12 NHS ester (ATTO-TEC GmbH, Siegen, Germany) is a lipophilic variant of the dye ATTO 655 with a molecular weight of 835 Da. Because of its positive charge ATTO-Oxa12 is an amphiphilic molecule that is soluble in water but also interacts with lipids. The spectral characterization of ATTO-Oxa12 is shown in Figure 7.

#### 3.1.2. Topical Dye Application to Skin Explants

All experiments were performed on intact excised human skin (upper arm), obtained within 6 h after excision from healthy volunteers undergoing plastic surgery after written informed consent according to the requirements by the ethic committee of the Charité-Universitätsmedizin Berlin (EA1/135/06, last renewal 2 May 2013) and in accordance with the Declaration of Helsinki Principles. Preexisting tissue damage was excluded macroscopically and microscopically prior to the experiments. Skin samples of 4 cm^2^ were wiped, 20 µL of 0.3 µM ATTO-Oxa12-solution or phosphate buffer (control) were applied onto the central 1 cm^2^ of the slightly stretched and fixed samples to avoid sideways non-specific penetration of the dye at the border of the tissue. After incubation in a humidified chamber at 37 °C, 5% CO_2_, and 100% humidity for 2 h, remaining superficial dye was carefully removed from the skin surface using cleaning wipes (Kimtech Science, Kimberly-Clark, Koblenz, Germany).

#### 3.1.3. Sample Preparation

Adhesive tape stripping was performed on a 1 cm^2^ skin area treated either with topically applied ATTO-Oxa12 or phosphate buffer. Adhesive transparent tapes (Tesafilm, Hamburg, Germany) were pressed onto the skin by a roller to minimize the influence of skin furrows and wrinkles and subsequently removed. This procedure was repeated at the same position up to 100 times. For TIRFM measurements the 5th, 6th, 10th and 20th stripped tapes were affixed air bubble-free on cleaned glass cover slips (170 ± 5 µm, 22 × 22 mm, No. 1.5H, Marienfeld, Lauda Königshofen, Germany) under clean bench conditions.

Longitudinal cryosections were obtained from the pretreated tissue blocks (see Section 3.1.2.). The sections were prepared as previously described [35] with a thickness of 5 µm.

### 3.2. Methods

#### 3.2.1. Fluorescence Microscopy

Screening experiments on the 100 removed adhesive tape strips were performed and in addition every 10th tape was analyzed by a conventional fluorescence microscope (AXIOPLAN microscope, Zeiss, Jena, Germany), equipped with a 40× objective. The samples were excited at 551 nm and the emission was detected above 573 nm. The mean fluorescence intensity of imaged corneocytes was analyzed by using an open source image processing program (ImageJ, NIH, Bethesda, MD, USA).

The cryosections were measured by confocal fluorescence microscopy (Laser scan microscope 700, Zeiss, Jena, Germany). Laser excitation of the samples was performed at 555 nm (10 mW) and the emission was detected using corresponding emission filters.

#### 3.2.2. Single Molecule TIRF Microscopy

**Figure 8:**
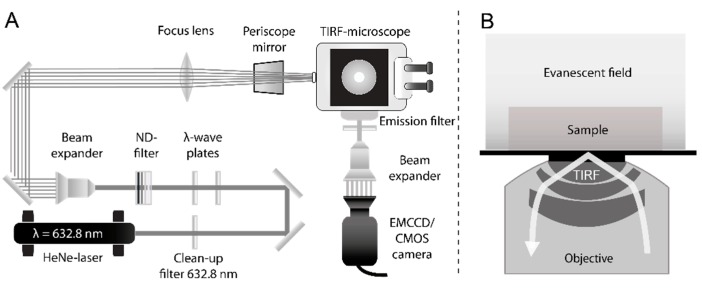
(**A**) Schematic of the TIRFM setup; (**B**) TIRF principle. Total internal reflection occurs at the glass cover slip surface generating an evanescent field, which decays with increasing distance from the surface. This leads to a small excitation layer above the glass slide, reducing background fluorescence. Sample and evanescent field are drawn schematically and are not to scale with the dimensions of the objective.

The TIRF microscopy setup used for single molecule measurements on tape strips and cryosections is shown in Figure 8. It consists of an inverted microscope (IX-71, Olympus, Hamburg, Germany), equipped with a 60× TIRF objective (numerical aperture (NA) = 1.45, PLAPON 60XOTIRFM, Olympus, Hamburg, Germany) [36,37,38,39]. Samples were excited by a helium-neon laser (λ = 623.8 nm, 21 mW, 1145P/JDS Uniphase, Milpitas, CA, USA). Before the laser light passes the microscope, neutral density filters adjust the excitation intensity and the initial polarization is changed from linear to circular polarization by consecutive λ/2 and λ/4 wave plates. A subsequent 10× beam expander (bm.x, Qioptiq, Göttingen, Germany) increases the beam diameter. The expanded beam is focused on the back focal plane of the TIRF objective by a plano-convex lens (750 mm, N-BK7, Thorlabs, Dachau, Germany) using the back port of the IX-71 and a periscope mirror. A dichroic beam splitter (z638RDC, Chroma, Bellows Falls, VT, USA) directs the emitted fluorescence into the left microscope side port where it passes a long pass filter (HQ 655LP, Chroma, Bellows Falls, VT, USA) to remove remaining laser light. The fluorescence emission is detected either by a CMOS (complementary metal-oxide semiconductor) camera (ORCA-Flash4.0 V2 C11440-22CU, Hamamatsu, Japan) or an EMCCD (electron multiplying charged coupled device) camera (C9100-13, Hamamatsu, Japan). The CMOS camera has a real time read out of up to 100 frames/s (10 ms integration time per frame) at its full resolution of 2048 × 2048 pixels and a readout noise of 1.6 electrons rms. Its cell size amounts to 6.5 µm^2^ resulting in a resolution of 106 nm per pixel width. The EMCCD camera has a real time read out of up to 32 frames/s (31 ms integration time per frame) at its full resolution of 512 × 512 pixels and a readout noise of 1.0 electrons. The cell size is 16 µm^2^. Therefore, the EMCCD camera is used in combination with a 3× beam expander (bm.x, Qioptiq, Göttingen, Germany) resulting in a resolution of 74 nm per pixel width. All measurements were recorded with the respective minimal integration time of either 10 ms (CMOS camera) or 31 ms (EMCCD camera), depending on the camera type.

#### 3.2.3. Single Molecule Data Analysis

**Figure 9:**
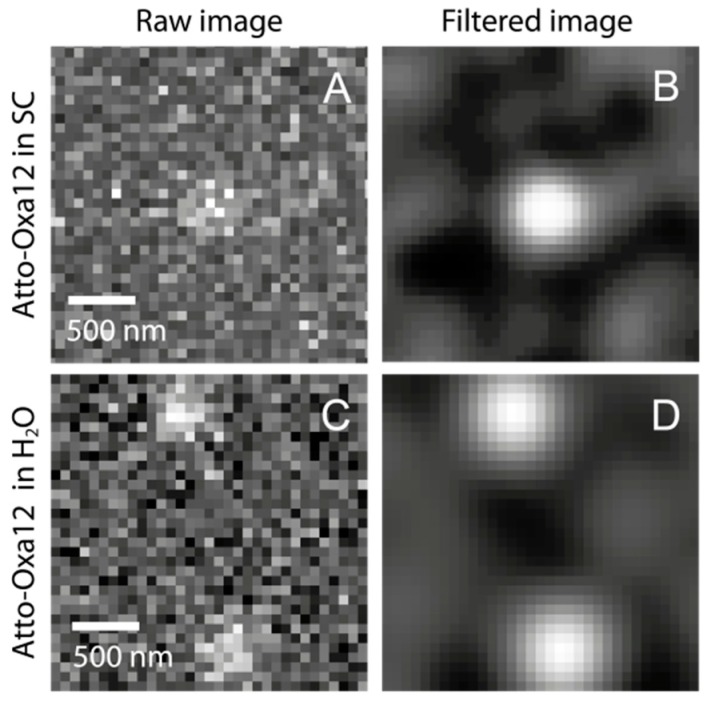
(**A**) TIRFM images of single Atto-Oxa12 molecules detected in the stratum corneum and in H_2_O: (**A**) individual raw image, showing an Atto-Oxa12 molecule in human SC; (**B**) Gaussian filtered image of (**A**); (**C**) Two single Atto-Oxa12 molecules in H_2_O are shown for comparison; (**D**) Gaussian filtered image of (**C**).

TIRFM movies were recorded using the Hamamatsu’s acquisition software (HiPic/HoKaWo, Hamamatsu, Japan) and saveds in 16-bit data format (HIS, Hamamatsu). To obtain the individual Atto-Oxa12 positions, a localization analysis was performed using the Localizer software package [40]. The single point spread functions (Figure 9) were approximated from the intensity profile of the ATTO-Oxa12 dye molecules with 2D Gaussian functions. The obtained molecule positions were used for diffusion analysis by both a trace-based mean square displacement (MSD) analysis and a histogram based step length (also called jump distance) analysis.

For the generation of step length distribution histograms using the statistical step length (jump distance) analysis approach all distances between molecule positions were sorted into a histogram by a self-written algorithm in Python, thus generating the step length distribution (SLD) over all molecules at a given lag time *t*_lag_ (*t*_lag_ is the product of frame index and frame interval, *i.e.*, the integration time of a single frame for the case at hand) [32,36,41]. To fit this distribution we used a probability density function describing the distribution of distances *r* between two random points in a circle (circular confinement) [42]. Here, we extended this function for multiple subpopulations:

(1) $$P_{\text{SLD}}\left(r\right)=\overset{N}{\underset{i}{\sum }}A_{i}\left(\frac{2r}{{a_{i}}^{2}}-\frac{4r\sin\left(\frac{r}{2a}\right)}{\pi \;{a_{i}}^{2}}-\frac{r^{2}\sqrt{4{a_{i}}^{2}-r}}{\pi \;{a_{i}}^{4}}\right)$$

*A_i_* are the amplitudes and *a_i_* the confinement radii of the *i*th subpopulation ranging from 1–*N.* A prerequisite for the use of Equation (1) is the prior knowledge of confined diffusion. This can be established by either fitting a general radial probability distribution [36,41] to the histograms obtained at various lag times generating MSDs or by analyzing the MSDs of individual traces (see below) for lag time dependence. Confined diffusion exists if there is no lag time dependence of the MSDs. The MSDs shown in Figure 5B were generated from the confinement values *a*, as in this case the squared confinement values *a*^2^ correspond to the MSD at a given lag time.

To obtain the MSDs of individual ATTO-Oxa12 molecules, the corresponding traces were determined via single particle tracking (SPT) using the Localizer software [40]. From the single molecule traces the MSD of a set of coordinates {*x_i_*, *y_i_*} was determined as the average over all overlapping pairs using the following equation:

(2) $$\text{MSD}=\frac{1}{f-q}\overset{f-q}{\underset{i=1}{\sum }}{\left(x_{i+q}-x_{i}\right)}^{2}+{\left(y_{i+q}-y_{i}\right)}^{2}$$

where *f* is the total number of frames, *q* the chosen frame lag (frame index) and *x_i_* and *y_i_* the particle coordinates along the image axes. For a two-dimensional free diffusion the diffusion coefficient *D* is related to the MSD by:

(3) $$\text{MSD}=4\;D\;t_{\text{lag}}$$

To obtain the confinement length *d* of the individual particle traces or for the subpopulations generated from the statistical analysis (see above), the MSDs were fitted with the time-dependent MSD function for a circular confinement [43]:

(4) $$\text{MSD}(\text{t})={\text{ a}}^{2}\;\left[1-8\;\sum_{n}\exp\left(-\frac{{\beta_{n}}^{2}D_{t_{lag}}}{a^{2}}\right)\frac{1}{{\beta_{n}}^{2}-1}\;\frac{{J_{0}}^{2}(\beta_{n})}{{J_{1}}^{2}(\beta_{n})}\right]$$

where *a = d*/2 is the confinement radius, *D* the diffusion coefficient and *J*_n_(*β_n_*) the Bessel functions of order *n.* The confinement lengths obtained for individual ATTO-Oxa12 traces were sorted into a histogram with a bin width of 18 nm. The visible populations in the histogram were fitted with the sum of Gaussian probability density functions.

## 4. Conclusions

Such detailed characterization of dynamical and structural parameters (diffusion constants and confinement of diffusion) of penetrating molecules within the different skin layers as obtained by single molecule TIRF microscopy improves our understanding of the penetration processes occurring in healthy and diseased skin and when designing successful transdermal drug delivery strategies. Not only diffusion constants can be directly obtained but also confinements, *i.e.*, sizes of penetration pathways in the nanometer range, are easily accessible. We performed the present study with a large amphiphilic model compound to show the feasibility of the single molecule approach. This now opens up the opportunity to conduct comparative studies of molecules with different sizes and polarities. Our studies demonstrate that conventional approaches to skin penetration can lead to under prediction of penetration as single penetrating molecules are not visible. Studies with focus on skin barrier restoration strategies, barrier-keratinocyte-immune cell interactions in the sub-corneal space as well as toxicological studies could benefit tremendously from the presented technology. Data obtained by such studies can be used to develop strategies which facilitate drug penetration by specifically targeting the SC compartment of interest for a specific molecule. Also, further investigation of differential penetration in diseased skin and skin disease models can help elucidate barrier function and possible consequences for drug penetration.
